# Testing for Serial Correlation in Autoregressive Exogenous Models with Possible GARCH Errors

**DOI:** 10.3390/e24081076

**Published:** 2022-08-04

**Authors:** Hanqing Li, Xiaohui Liu, Yuting Chen, Yawen Fan

**Affiliations:** 1 School of Statistics, Jiangxi University of Finance and Economics, Nanchang 330013, China; 2 Key Laboratory of Data Science in Finance and Economics, Jiangxi University of Finance and Economics, Nanchang 330013, China; 3 College of Behavioral and Social Sciences, University of Maryland, College Park, MD 20742, USA

**Keywords:** autocorrelation, serial correlation, empirical likelihood, ARX model, C12, C22

## Abstract

Autoregressive exogenous, hereafter ARX, models are widely adopted in time series-related domains as they can be regarded as the combination of an autoregressive process and a predictive regression. Within a more complex structure, extant diagnostic checking methods face difficulties in remaining validity in many conditions existing in real applications, such as heteroscedasticity and error correlations exhibited between the ARX model itself and its exogenous processes. For these reasons, we propose a new serial correlation test method based on the profile empirical likelihood. Simulation results, as well as two real data examples, show that our method has a good performance in all mentioned conditions.

## 1. Introduction

Time series data are frequently encountered in fields like weather forecasting, earthquake prediction, and electroencephalography. How to analyze time series data has been of great interest in statistics for a long time. Many famous models have been developed to study the relationships in time series data. Univariate time-series models include autoregressive models (AR), moving average models (MA), autoregressive moving average (ARMA), predictive regression models (PRM), and so on. Extensions of these models to handle vector-valued data contain, e.g., vector autoregression models; see, for example, ref. [1] for detailed discussions of these models.

Among others, the AR model, firstly proposed by [2], takes the simplest form. It specifies that the output variable only depends linearly on its own previous values and the random error term. But in many situations, auxiliary information is available and can be treated as covariates for modeling. Furthermore, the inclusion of auxiliary variables can improve the estimation efficiency. Hence, the so-called autoregressive exogenous (ARX) model was developed in previous literature to incorporate this benefit. The ARX model takes the following form:

(1) $$I\left(B\right)Y_{t}=\alpha +\beta^{⊤}X_{t-1}+U_{t},$$

where $Y_{t}$ denotes the response, $I\left(B\right)=1-\sum_{i=1}^{p}a_{i}B^{i}$, and *B* denotes the backshift operator with $B^{l}Y_{t}=Y_{t-l}$ for l≥1. Suppose $X_{t}$ is the covariate that contains useful auxiliary information. $U_{t}$s are model errors with means of 0 and finite variances. Without confusion, we assume that $\left\{X_{t}\right\}$ follows the first-order vector autoregressive (VAR) model:

(2) $$X_{t}=\mu +AX_{t-1}+V_{t},$$

where $V_{t}$ denotes the vector-valued random errors. α, β, μ and A are unknown parameters.

Moreover, the ARX model (1) has been widely adopted to analyze time series data in many fields. For example, in finance, as narrated in [3], an illustration of (1) can be that $Y_{t}$ reflects a change in an asset’s price, and $X_{t}\in R^{d}$ are lagged variables related to asset prices. A more specific example is that [4] employed the VARX model to investigate the relationship between the closing price of HRUM energy and PTBA (endogenous variable) and the exchange rate (exogenous variable). Ref. [5] applied the ARX model in the generalized space and used the international crude palm oil (CPO) prices as exogenous variables to predict the export volume of CPO. Additionally, in the environmental area, Ref. [6] analyzed hourly ozone data collected routinely at several monitoring sites in Austria using different ARX models to complete a pollution assessment. Furthermore, Ref. [7] used ARX-GARCH models to forecast air quality levels using daily data from the monitoring stations of 16 cities/counties in southeast China.

Some pre-test procedures are needed when specifying time series like (1), such as testing the existence of unit root in series $Y_{t}$. To achieve this, there is a useful technique of replacing I(B) in (1) by a specific linear filter $P\left(B\right)=1-\phi B-\sum_{i=1}^{p-1}\psi_{i}\Delta^{i}$, in which Δ denotes the difference operator. Note that $\phi =\sum_{i=1}^{p}a_{i}$ and $\psi_{i}=-\sum_{j=i}^{p-1}a_{j+1}$ for i=1,⋯,p−1. Hence, we can rewrite (1) in the following form (3). This trick is similar to the so-called Dickey–Fuller reparameterization [8,9], which is proposed for constructing a Dickey–Fuller unit root test.

(3) $$Y_{t}=\alpha +\phi Y_{t-1}+\sum_{i=1}^{p-1}\psi_{i}\Delta Y_{t-i}+\beta^{⊤}X_{t-1}+U_{t}.$$

The reparameterization trick given in (3) is most notable for its convenience of using ϕ instead of $\sum_{i=1}^{p}a_{i}$ to represent the stationarity property of the endogenous structure of $Y_{t}$. Hence, we choose (3) as the expression of our ARX model in the following discussions.

Owing to its wide applications, many researchers have focused on the theory of model specification of ARX models, including both parametric and nonparametric methods. To name but a few, ref. [10] proposed a least squares method to estimate the parameters in linear and nonlinear ARMAX models and discussed the asymptotic properties of these estimators. Ref. [11] employed a local polynomial fitting scheme incorporated with projections to obtain nonparametric estimation of additive nonlinear ARX time series. Ref. [12] introduced a quasi-likelihood method in estimating a censored ARX model and proved the quasi-likelihood estimation computationally efficient; see, e.g., [12] and references therein for more details on this topic.

Despite the fact that numerous studies have investigated different methods to specify and estimate the ARX model, little literature has discussed the issue of testing its serial correlation. Most studies assume that errors are of no autocorrelations; however, this assumption can be often violated, and it may negatively affect our further inference. For instance, the endogeneity problem, as a common cause of serial correlation, may destroy the consistency of the regression coefficients, leading to misspecification of the model, and damage the explanatory properties in real applications. Therefore, it is of importance to check for serial correlation of the random model errors.

There are many methods of testing serial correlations based on the least squares (LS) residuals. Along this direction, Ref. [13] proposed the first corresponding test procedure, the Durbin–Watson (DW) test. Unfortunately, it only works in testing the first-order autocorrelation. To deal with this limitation, the portmanteau test constructed by *Q* statistics gained great popularity; this test is also known as the *Q* test. The two most famous forms of *Q* tests are the Box–Pierce (BP) and Ljung–Box (LB) tests, proposed in [14,15], respectively. However, much evidence shows that *Q* tests suffer from the size distortion issue for models with AR structures; see, e.g., [16], who argued against the validity of *Q* tests by showing that the asymptotic chi-square distribution under the null hypothesis of no autocorrelations relies on the strictly exogenous nature of all regressors to the error terms. Although some further works like [17,18] expanded the adoption of portmanteau tests to ARMA models by developing the asymptotic results of *Q* statistics, their techniques cannot apply to the ARX models since the combination of AR and exogenous structure leads to an increase in complexity.

Another alternative is to use the plug-in (PI) empirical likelihood method, which follows the approach in [19], or the Breusch—Godfrey (BG) test from [20,21]. However, it is worth noting that in practice, such as the financial data discussed in [3], the $U_{t}$ in (3) and $V_{t}$ in (2) might be correlated. This correlation could result in a biased LS estimator when facing a finite sample size. This means that the plug-in empirical likelihood method fails to work in such a situation. For the BG test, although it seems to perform stably against the possible correlation existing between $U_{t}$ and $V_{t}$ (as will be reported in our simulations), there are still some more scenarios for which the BG test cannot be applied. For instance, the heteroscedasticity mentioned above can violate its disturbance assumptions. Consequently, it not only leads to inefficient parameter estimations, but also breaks the conditions for the application of the BG testing method that relies on LS residuals.

Note that the ARX models are often used in financial data analysis, in which volatility clustering often occurs. Ref. [22] firstly noted this phenomenon. From his description, volatility clustering is defined by the idea that large changes tend to be followed by large changes of either sign, and small changes tend to be followed by small changes. A number of following studies focused on the impacts of its existence in real applications; see, e.g., discussions in [23,24,25]. To address such a feature, a family of widely adopted models named autoregressive conditional heteroscedasticity (ARCH) and generalized autoregressive conditional heteroscedasticity (GARCH) have been proposed, respectively, by [26,27]. With the conditional serial dependence structures, known as conditional heteroscedasticity, economists have found a more appropriate approach to modeling data than following the obviously false assumption of homoscedasticity.

It is interesting to find that when dealing with GARCH errors, many existing serial correlation tests, including the LB, BG, and plug-in empirical likelihood method, suffer from size distortion; see our simulation in the sequel.

To deal with this problem, we construct a new test for the ARX model based on the profile empirical likelihood method motivated by [28]. It turns out that the proposed test for serial correlations performs robustly in the heteroscedastic as well as correlated $U_{t}$ and $V_{t}$ situations.

The rest of this paper is organized as follows. In Section 2, we present the serial correlation test and the main results. Section 3 reports the finite sample performance of the proposed testing statistic. Section 4 further applies the test to financial data and environmental data. Section 5 concludes the whole paper. Detailed proofs of the main results can be found in Appendix A.

## 2. Methodologies and Main Results

Assume that ${\left\{Y_{t},X_{t-1}\right\}}_{t=1}^{n}$ are generated from (2) and (3). Our aim is to construct a test method to check whether serial correlations exist in ${\left\{U_{t}\right\}}_{t=1}^{n}$. The hypothesis of interest is as follows:

$$H_{0}:\gamma =0\;↔\;H_{1}:\gamma \neq 0,$$

i.e., to check whether there exist *q*-th serial correlations in the model errors, where $\gamma_{k}=E\left(U_{t}U_{t-k}\right)$ and $\gamma ={\left(\gamma_{1},\cdots ,\gamma_{q}\right)}^{⊤}$ for some positive integer *q*.

Since the definition of γ involves the expectation, a straightforward idea is to construct some testing statistics for $H_{0}$ by using the plug-in (PI) empirical likelihood method; that is, plugging the LS residuals of $U_{t}$ in the constraints of empirical likelihood ratio function. The details are as follows: define $\theta ={\left(\alpha ,\phi ,\psi^{⊤},\beta^{⊤}\right)}^{⊤}$, and denote the LS estimate of θ as $\hat{\theta }:={\left(\hat{\alpha },\hat{\phi },{\hat{\psi }}^{⊤},{\hat{\beta }}^{⊤}\right)}^{⊤}$; then, the residuals ${\left\{{\hat{U}}_{t}\right\}}_{t=p+q+1}^{n}$ can be easily obtained. Write $t_{0}=p+q+1$ and N=n−p−q. Then, the PI method is obtained by calculating the following EL ratio function as

(4) $$L\left(\gamma \right)=\sup\left\{\prod_{t=t_{0}}^{n}Np_{t}:p_{t}\geq 0,\sum_{t=t_{0}}^{n}p_{t}=1,\sum_{t=t_{0}}^{n}p_{t}Z_{t}\left(\gamma \right)=0\right\},$$

where $Z_{t}\left(\gamma \right)={\left({\hat{U}}_{t}{\hat{U}}_{t-1}-\gamma_{1},\cdots ,{\hat{U}}_{t}{\hat{U}}_{t-q}-\gamma_{q}\right)}^{⊤}$ with ${\hat{U}}_{t}=Y_{t}-\hat{\alpha }-\hat{\phi }Y_{t-1}-\sum_{i=1}^{p-1}{\hat{\psi }}_{i}\Delta Y_{t-i}-{\hat{\beta }}^{⊤}X_{t-1}$.

Following a similar proof to that of [19], it is possible to show that −2logL(0) is asymptotically chi-square distributed once $\left\{U_{t}\right\}$ is a sequence of independent and identically distributed errors under some mild conditions. Hence, for given observations ${\left\{Y_{t},X_{t-1}\right\}}_{t=1}^{n}$, one may reject the null hypothesis once $-2\log L\left(0\right)>\chi_{q}^{2}\left(1-\tau \right)$ at the significance level τ∈(0,1), where $\chi_{q}^{2}\left(1-\tau \right)$ denotes the (1−τ)-th quantile of the chi-squared distributed variable with *q* degree of freedoms.

However, although this method is computationally fast and can avoid the variance estimation, it still suffers from a strong undersized distortion. As an improvement, we may treat θ as redundant parameters, and, similar to [28], construct a series correlation test by using the profile empirical likelihood (PEL) method.

In detail, first define ${\left\{U_{t}\left(\theta \right)\right\}}_{t=t_{0}}^{n}$ where

(5) $$U_{t}\left(\theta \right)=Y_{t}-\alpha -\phi Y_{t-1}-\sum_{i=1}^{p-1}\psi_{i}\Delta Y_{t-i}-\beta^{⊤}X_{t-1}.$$

Then, similar to [19], an empirical likelihood function for the unknown θ and γ can be defined as follows:

(6) $$\tilde{L}\left(\theta ,\gamma \right)=\sup\left\{\prod_{t=t_{0}}^{n}Np_{t}:p_{t}\geq 0,\sum_{t=t_{0}}^{n}p_{t}=1,\sum_{t=t_{0}}^{n}p_{t}{\tilde{Z}}_{t}\left(\theta ,\gamma \right)=0\right\},$$

where ${\tilde{Z}}_{t}\left(\theta ,\gamma \right)={\left({\tilde{Z}}_{t,1}\left(\theta ,\gamma \right),{\tilde{Z}}_{t,2}\left(\theta ,\gamma \right),\cdots ,{\tilde{Z}}_{t,p+d+q+1}\left(\theta ,\gamma \right)\right)}^{⊤}$ with

$$\begin{array}{c} \left\{\begin{array}{ccc} {\tilde{Z}}_{t,1}\left(\theta ,\gamma \right) & = & U_{t}\left(\theta \right),\\ {\tilde{Z}}_{t,2}\left(\theta ,\gamma \right) & = & U_{t}\left(\theta \right)Y_{t-1},\\ {\tilde{Z}}_{t,j+2}\left(\theta ,\gamma \right) & = & U_{t}\left(\theta \right)\Delta Y_{t-j},\;\;j=1,2,\cdots ,p-1,\\ {\tilde{Z}}_{t,p+l+1}\left(\theta ,\gamma \right) & = & U_{t}\left(\theta \right)X_{t-1,l},\;\;l=1,2,\cdots ,d,\\ {\tilde{Z}}_{t,p+d+k+1}\left(\theta ,\gamma \right) & = & U_{t}\left(\theta \right)U_{t-k}\left(\theta \right)-\gamma_{k},\;\;k=1,2,\cdots ,q. \end{array}\right. \end{array}$$

Here, $X_{t-1,l}$ denotes the *l*-th component of $X_{t-1}$ for l=1,2,⋯,d.

Since we are interested in γ, we may eliminate the effect of θ by the profile method, as did [28]. The resulting profile empirical likelihood function L(γ) for γ can be defined as follows:

(7) $$L\left(\gamma \right)=\underset{\theta }{\sup}\tilde{L}\left(\theta ,\gamma \right).$$

To facilitate studying the limiting distribution of L(γ), we need to first specify the following assumptions:

- **A1**. $\left\{X_{t}\right\}$ are a strictly stationary sequence with the initial value $X_{0}$ being a constant vector.
- **A2**. ${\left\{U_{t},V_{t-1}\right\}}_{t=1}^{n}$ are Martingale difference sequences to the sigma field $F_{t}=\sigma \left(U_{s},V_{s}:s\leq t\right)$.
- **A3**. $E\left(U_{t}^{2}\right)=\sigma_{U}^{2},E\left(V_{t,1}^{2}\right)=\sigma_{V_{1}}^{2},\cdots ,E\left(V_{t,d}^{2}\right)=\sigma_{V_{d}}^{2}$ almost surely.
- **A4**. $\sup_{t\geq 1}E(|U_{t}{|}^{2+\delta }+|V_{t,1}{|}^{2+\delta }+\cdots +|V_{t,d}{|}^{2+\delta })<\infty$ for some δ>0 almost surely.
- **A5**. |ϕ|<1 and all roots of $1-\sum_{i=1}^{p-1}\psi_{i}x^{i}=0$ are inside the unit circle.

Hereafter, $F_{t}$ denotes the information set available at time t∈{1,2,⋯,n}. Based on these assumptions, we can obtain our main result of Theorem 1.

**Theorem** **1.** *Suppose assumptions* ***A1**–**A5**hold. Then, under the null hypothesis $H_{0}$, we have*

$$\begin{array}{c} -2\log L\left(\gamma \right)\overset{d}{⟶}\chi_{q}^{2}, \end{array}$$

*as n→∞, where $\chi_{q}^{2}$ denotes the chi-squared distributed variable with q degrees of freedom, and $\overset{d}{⟶}$ denotes the convergence in distribution.*

Theorem 1 is desirable because there is no need to estimate the asymptotic variance, which is difficult especially when the model errors follow the GARCH process. In practice, we may reject the null hypothesis $H_{0}$ if

$$-2\log L\left(0\right)>\chi_{q}^{2}\left(1-\tau \right)$$

at the significance level τ∈(0,1), analyzing the random observations ${\left\{Y_{t},X_{t-1}\right\}}_{t=1}^{n}$.

## 3. Simulation Studies

In this section, we conduct simulations to investigate the performance of our proposed testing method, i.e., the PEL test for the ARX model. Meanwhile, we compare the testing with the other two extant testings, including the PI and BG tests. Firstly, we set two processes in generating $U_{t}$, and let ${\left\{e_{t}\right\}}_{t=1}^{n}$ be an independent identically distributed standard normal series with $e_{0}=0$. The two forms of $U_{t}$ are, respectively,

(8) $$\begin{array}{ccc} & U_{t}=\lambda U_{t-1}+e_{t}, & \end{array}$$

(9) $$\begin{array}{ccc} & U_{t}=\eta_{t}\sigma_{t},\;\sigma_{t}^{2}=0.01+0.32U_{t-1}^{2}+0.23\sigma_{t-1}^{2},\;\eta_{t}=\frac{\lambda e_{t-1}+e_{t}}{\sqrt{1+\lambda^{2}}}. & \end{array}$$

The autocorrelation of $U_{t}$ in (9) is from the simple AR(1) structure generated by nonzero λ, and (9) as a GARCH(1,1) process depicts a relatively complex heteroscedastic structure. When λ=0, the results refer to the test sizes. To examine the local powers, we then set $\lambda =1/\sqrt{n},\cdots ,4/\sqrt{n}$.

The correlations between $U_{t}$ and $V_{t}$ are designed by $\rho ={\left(\rho_{1},\cdots ,\rho_{d}\right)}^{⊤}$. This vector determines the variance matrix of the joint normal distribution of $U_{t}$ and $V_{t}$ in which we assume

$$\left(\begin{array}{c} e_{t}\\ V_{t} \end{array}\right)∼N\left(0,\Gamma \right),\;\Gamma =\left[\begin{array}{cccccc} 1 & \rho_{1} & \rho_{2} & \cdots & \rho_{d-1} & \rho_{d}\\ \rho_{1} & 1 & 0 & \cdots & 0 & 0\\ \rho_{2} & 0 & 1 & \cdots & 0 & 0\\ \vdots & \vdots & \vdots & \ddots & \vdots & \vdots \\ \rho_{d-1} & 0 & 0 & \cdots & 1 & 0\\ \rho_{d} & 0 & 0 & \cdots & 0 & 1 \end{array}\right],$$

Obviously, $\mathrm{Cov}\left(U_{t},V_{t,k}\right)=\rho_{k}$ for 1≤t≤n,1≤k≤d, and $\mathrm{Cov}(V_{t,k},V_{t,l})=0$ for 1≤t≤n,1≤k≠l≤d.

For convenience, we consider the ARX model in the following form:

(10) $$\begin{array}{c} \left\{\begin{array}{c} Y_{t}=\alpha +\phi Y_{t-1}+\psi \Delta Y_{t-1}+\beta X_{t-1}+U_{t},\\ X_{t}=\mu +AX_{t-1}+V_{t}. \end{array}\right. \end{array}$$

The intercept and slope coefficient of $X_{t}$ were set as constants; that is, μ=0.01 and β=2.14. The slope of difference term $\Delta Y_{t-1}$ is ψ=0.13. There are two settings of α, 0 and 0.01, to imitate the presence or absence of an intercept. For both ϕ and *A*, we considered two conditions, 0.6 and 1, in which 0.6 refers to the stationary condition, and 1 refers to the unit root process. The covariance of $U_{t}$ and $V_{t}$, i.e. ρ, was set to 0,0.4,0.8, representing no correlation, light correlation and heavy correlation, respectively. The sample size *n* was 200 and 800. For each case, we generated 10,000 random samples.

**Figure 1:**
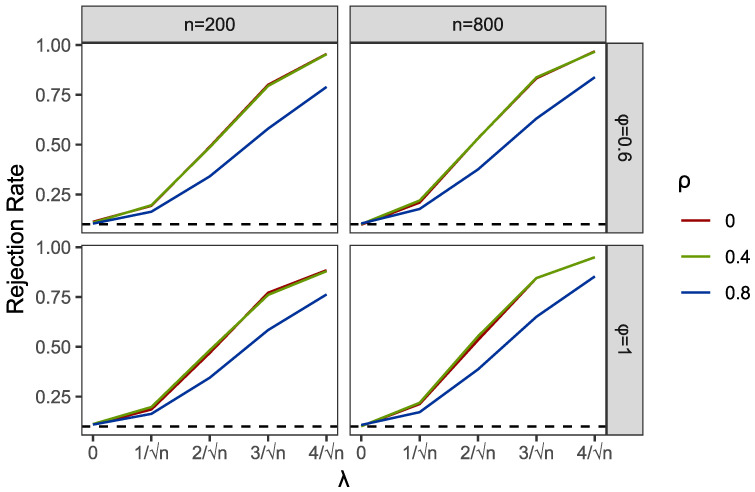
Empirical rejection rates of PEL test in AR structural $U_{t}$ with α=0 and A=0.6.

**Figure 2:**
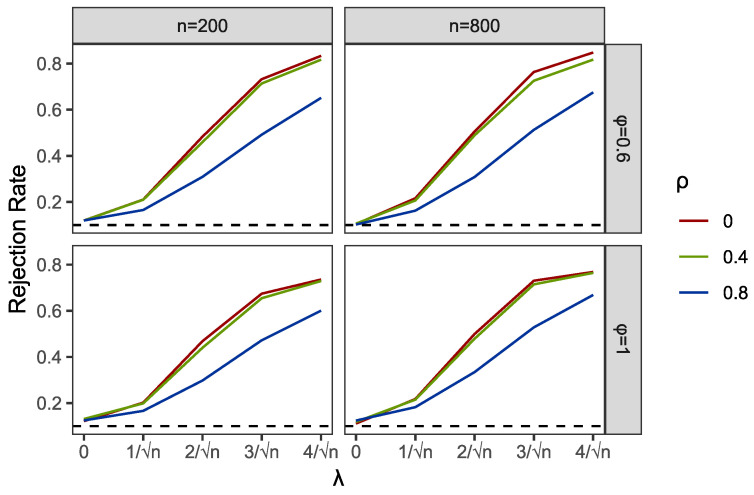
Empirical rejection rates of PEL test in GARCH structural $U_{t}$ with α=0 and A=0.6.

**Figure 3:**
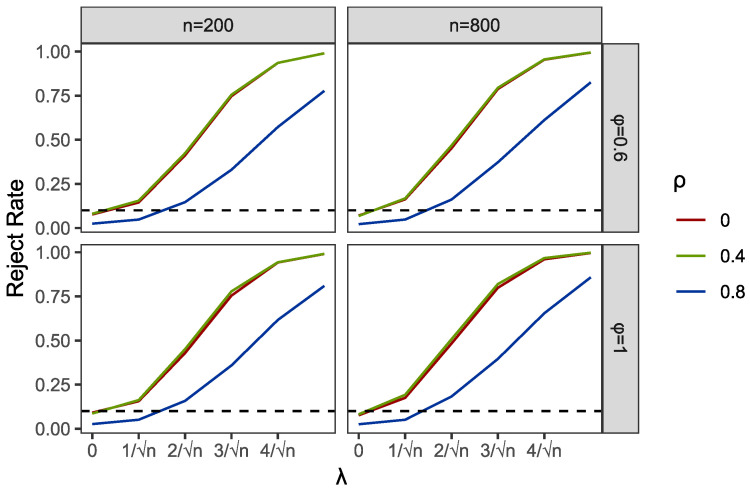
Empirical rejection rates of PI test in AR structural $U_{t}$ with α=0 and A=0.6.

**Figure 4:**
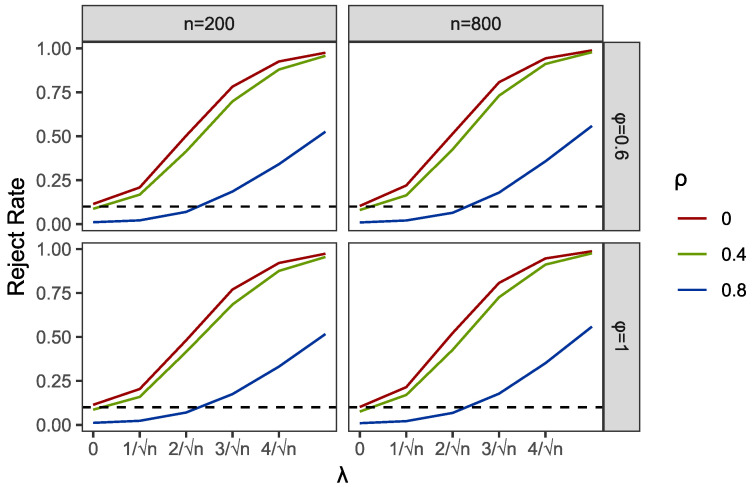
Empirical rejection rates of PI test in GARCH structural $U_{t}$ with α=0 and A=0.6.

**Figure 5:**
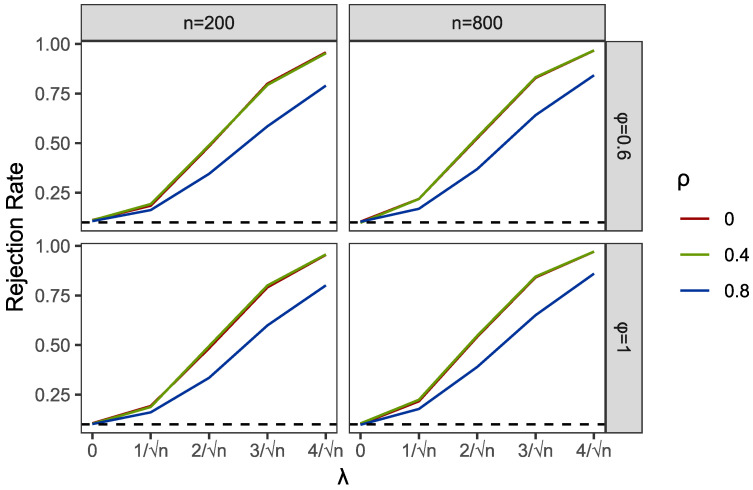
Empirical rejection rates of BG test in AR structural $U_{t}$ with α=0 and A=0.6.

**Figure 6:**
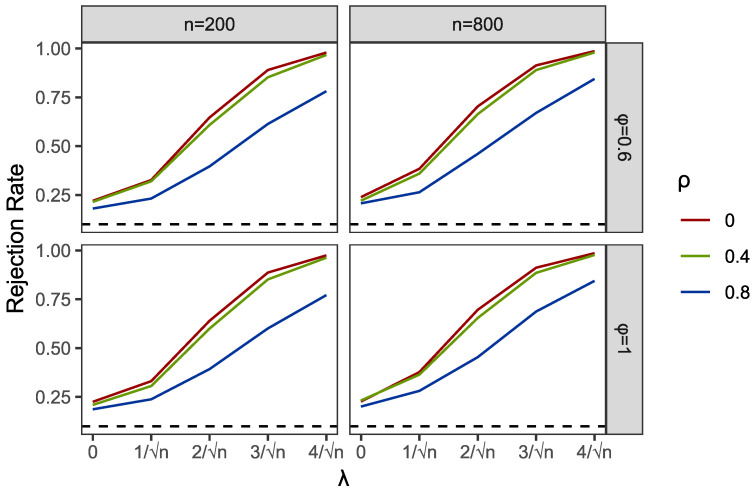
Empirical rejection rates of BG test in GARCH structural $U_{t}$ with α=0 and A=0.6.

Within the programming of profile empirical likelihood function in the PEL and PI tests, we used R functions “el.test” in the package “emplik”. Meanwhile, the R function “nlm” was applied to complete the profile optimization in PEL. We used the R function “bg.test” to obtain the results of the BG test. Then, we reported the comparisons of empirical sizes and powers of PEL, PI and BG in Table 1 and Table 2 and Figure 1, Figure 2, Figure 3, Figure 4, Figure 5 and Figure 6.

The results of the empirical sizes and powers of the AR structural $U_{t}$ are shown in Table 1. As we expected, our proposed PEL test performs well in all assumed conditions. While in some settings, the simulation with smaller sample size n=200 shows a slight oversized distortion, e.g., when both ϕ and *A* are 1 (nonstationary AR structures of $X_{t}$ and $Y_{t}$), as the sample size increases to n=800, the sizes all converge to the presumed τ. Note that there are some methods in literature that can be used to adjust the size of the empirical likelihood method when the sample size is relatively small. One may improve the size performance by the Bartlett correction; see, e.g., [29] for details. Furthermore, from Table 1, we can see that with the AR structural $U_{t}$, the BG test also works well, and it is efficient regardless of the presence of a strong correlation between $U_{t}$ and $V_{t}$. However, for the PI tests, the results indicate that the corresponding sizes suffer from obvious undersized distortions due to the autoregressive structure. Similar to portmanteau *Q* tests such as BP and LB, the PI test has the constraint that the regressors should be exogenous to avoid the disturbance from the plug-in estimators of the autoregressive coefficients, so PI is not suitable for ARX models.

Results of empirical sizes and powers of the GARCH structural $U_{t}$ are shown in Table 2. Different from the cases with AR structural $U_{t}$, the sizes of the BG test are highly deviated from the presumed τ, which means the heteroscedastic structure of random disturbances completely destroys the validity of the BG test. However, the PI test works well in some settings under GARCH-type errors. This is also shown in Figure 4. For instance, when $U_{t}$ is not or weakly correlated with $V_{t}$, such as ρ=0 and 0.4, the results of the PI test are similar to the size values τ given in advance. However, when the correlations increase or even close to 1, which often occurs in practice, the PI test fails and shows a strong undersized distortion. Compared to both the BG and PI tests, the PEL method shows its robustness. Note that only with the smaller sample size n=200, the sizes of PEL tests show notable overrejections, but these distortions mostly disappear with the increase in sample sizes; see the results of n=800. Except in a special condition when $X_{t}$ and $Y_{t}$ are both nonstationary(ϕ=A=1), and $U_{t}$ is extremely strongly associated with $V_{t}$, we can find a non-negligible overrejection by comparing the sizes of PEL with the predetermined sizes τ, even at a sample size of 800.

Part of the simulation results are plotted in Figure 1, Figure 2, Figure 3, Figure 4, Figure 5 and Figure 6. Comparing the first two figures which show the results of the PEL test in AR and GARCH errors, the increasing speeds of rejection rates of GARCH errors vary with λ and are slower than those of AR errors. Nevertheless, the PEL test remains effective with GARCH errors. From the next two figures, we can see a good performance of PI test in limited endogenous GARCH errors, but it fails when the endogenous errors are too strong. The results of PI testing in AR-type errors also indicate apparent distortions. Finally, in the last two figures, we can find that the BG test performs well in AR errors but becomes invalid with respect to GARCH errors, as discussed above.

## 4. Real Applications

This section is devoted to applying the proposed PEL test to examining the error autocorrelations in two real applications from the financial and environmental fields. We then compare the findings with the results from the other two testing methods, the PI and BG methods.

### 4.1. A Financial Example

In last few decades, numerous studies have investigated whether stock returns can be predicted by financial and economic variables, such as the dividend–price ratio, the earnings–price and other measures of the interest rate. For instance, ref. [30] implemented a test of predictability on U.S. equity data. They indeed found reliable evidence for predictability with the earnings–price ratio, the T-bill rate, and the yield spread on stock returns, and weaker evidence for predictability with the dividend–price ratio. In addition, ref. [17,18] concluded that an ARMA(1,1) model of the stock returns cannot be rejected. Therefore, the stock returns at lag 1 might be a potential explanatory variable.

**Figure 7:**
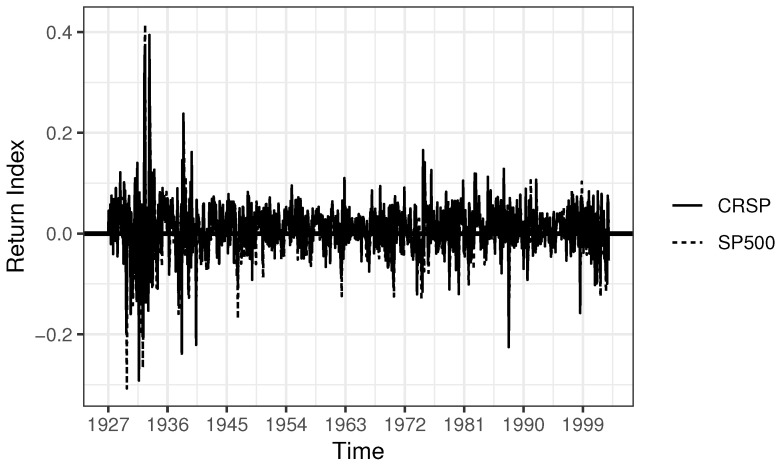
Tendencies of two stock return indexes.

In this subsection, we apply the new proposed PEL method to revisit U.S. equity data analyzed by [30]. More specifically, two series of stock returns are involved in the empirical study, monthly S&P 500 index data from Global Financial Data and monthly CRSP value-weighted index data from the Center for Research in Security Prices (CRSP). See Figure 7.

Similar to [30], stock return indexes were regarded as the predicted variable, and three different sample periods were considered: 1927–2002, 1927–1994 and 1952–2002. The reasons for splitting the sample period are that around 1994, valuation ratios drifted to historical lows, making the process more nonstationary, and the nature of U.S. interest rates was changed by the Fed’s pegging rate policy after 1952. For the first two periods, 1927–2002 and 1927–1994, we only included two exogenous variables, log earnings–price (lep) ratio and log dividend–price (ldp) ratio. The earnings–price ratio was computed as a moving average of earnings over the past ten years divided by the current price, and the dividend–price ratio is computed as dividends over the past year divided by the current price. For the sample period 1952–2002, this allowed us to add two additional exogenous variables, the treasury-bill (tbl) rates and the long yield (lty) spread. The short rate was the one-month treasury-bill rate, and the long yield spread was Moody’s seasoned Aaa corporate bond yield. Finally, the stock returns at lag 1 were treated as another predictor among all sample periods.

Firstly, we fitted the same predictive regression models of the monthly data as [30] for the three different sample periods and applied PEL testings to examine serial correlation. The *p*-values are reported in Table 3. Even though [30] provided some evidence about the predictability of log earnings-price ratio on stock returns for the subsample periods 1927–2002 and 1927–1994, our PEL testings indicate that we should reject the null hypothesis of no higher-order serial correlations at the 10% level of significance. Therefore, it might be necessary to add further model correction steps when forecasting the stock returns.

**Figure 8:**
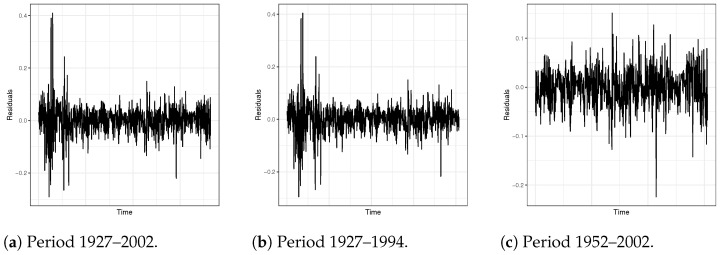
Residuals of ARX models.

Next, besides the multiple econometric variables mentioned above, we also considered the stock returns at lag 1 as predicting variables in ARX models. Then, PEL and BG testings were applied for diagnostic checking, respectively. All results are reported in Table 4. From Table 4, we cannot reject the null hypothesis at the 5% significance level based on the results from PEL testings for all sample periods. The PI testings indicate the same conclusions as the PEL testings at the 5% level of significance. However, the corresponding *p*-values seem to be extremely huge. This could be because the high correlations between $U_{t}$ and $V_{t}$ make the PI testings invalid: the Pearson’s correlation coefficients from all three periods are greater than 0.95. For the BG testings, the results provide the opposite conclusions when looking at sample periods 1927–2002 and 1927–1994. The conclusions show that the ARX models are not adequate. However, for the subsample period 1952–2002, BG testing gives evidence that we should not reject the null hypothesis of no serial correlation at the 5% level of significance. The reason for the different evidence is that BG testing could be invalid when the dataset suffers from heteroscedasticity problems, like the sample periods 1927–2002 and 1927–1994 (see Figure 8). This is consistent with our simulation studies. Furthermore, compared to the PEL testing proposed in this paper, the BG testing might be more vulnerable to over-fitting.

### 4.2. An Environmental Example

**Figure 9:**
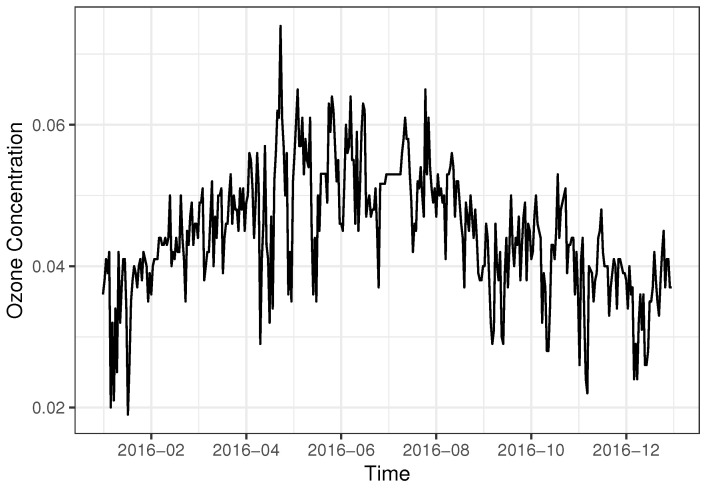
Tendency of ozone concentration.

As mentioned before, ARX models are also popular in the area of environmetrics. A typical usage is in pollution assessment. In this subsection, we use the ARX model to analyze daily ozone data in 2016, which are collected by a monitoring station in Texas, USA. See Figure 9. The ozone dataset can be downloaded from United States Environmental Protection Agency (EPA). Meteorological conditions can strongly affect the rates and completeness of the reactions producing ozone, as well as the subsequent transport and depletion; see [31]. Therefore, in line with [6], the set of exogenous variables includes air temperature, wind speed, extraterrestrial radiation and relative humidity. The meteorological data were collected by the USBA-ARS Conservation and Production Research Laboratory located in Bushland, Texas.

The ozone dataset contains some missing values from 27 June 2016 to 10 July 2016. Rather than deleting these dates or simply using means to impute, we split the dataset into two series: 1 January 2016–26 June 2016 and 11 July 2016–31 December 2016. In [6], they analyzed hourly data points and suggested lags greater than 48 for the autoregressive part. In the current study, the dataset is in daily frequencies, and so we chose ozone at lag 1 and lag 2 as predictors as well.

Then, we applied the three different testings, PEL, PI and BG testings, on the specified ARX models separately. All results are reported in Table 5. From Table 5, at least for the lower-order serial correlations, there is no reliable evidence for the presence of serial correlation for the subsample period 1 January–26 June at the 5% significance level. This means the ARX model is adequate. However, for the subsample period 11 July–31 December, the results of PEL testing indicate that we should reject the null hypothesis of no existence of error autocorrelations at the 5% significance level. The findings from PEL testings are consistent with the findings of [6] that global ARX models might be not adequate and it is necessary to consider other advanced models, such as ARXd and ARXr models. However, for the two subsample periods, all results of PI testings provide evidence that we should not reject the null hypothesis at the 5% significance level. This indicates the global ARX models are adequate, which contradicts the findings of previous studies. We then conducted the Pearson’s correlation tests and found that we should reject the null hypothesis of the zero correlations between $U_{t}$ and $V_{t}$ at the 5% significance level. Therefore, it is confirmed that the PI method are prone to the underfitting problem when $U_{t}$ and $V_{t}$ are correlated. When looking at the results from BG testings, we can see more sophisticated results. When q=2, it indicates we should reject the null hypothesis of no serial correlation at the 5% level of significance. However, as *q* increases to greater than 4, the serial correlation seems to disappear at the 5% level of significance.

## 5. Conclusions

In this paper, we develop a new test procedure for serial correlation in ARX models based on the profile empirical likelihood method in [28] and propose that the testing procedure holds even when the innovations of disturbances are heteroscedastic or the error series of ARX models and the exogenous processes are correlated. In the simulation studies, the PEL testing performs well in these conditions.

Furthermore, we analyze two real data in financial and environmental domains by specifying ARX models and then use the PEL testing method for model diagnosis. The first application shows that when using the PEL testing method, the first-order autoregressive models with exogenous variables, such as log earning-price and log dividend-price, are useful and appropriate in interpreting stock returns. In the second application, we examined whether the first-order or second-order autoregressive model with four exogenous variables, including air temperature, wind speed, extraterrestrial radiation and relative humidity, is appropriate for forecasting the ozone concentrations. The results indicate the pollution assessment could more complex and we should consider more advanced models.

In addition, our simulations and real applications mentioned possible nonstationary models, including unit root or nearly unit root processes. The discussions about the related theory and real examples can be seen in [32,33,34], etc. However, since (A4) in Lemma A2 does not hold, as (A5) does not converge in probability when ϕ=1, the rigorous proofs of the chi-squared distribution of proposed EL statistic in nonstationary situations are not given. We seek to address this issue in the future.

## Figures and Tables

**Table 1:** Empirical sizes and powers of AR structural $U_{t}$.

(ρ,	ϕ,	A,	λ)	α=0						α=0.01					
				n=200			n=800			n=200			n=800		
				**PEL**	**PI**	**BG**	**PEL**	**PI**	**BG**	**PEL**	**PI**	**BG**	**PEL**	**PI**	**BG**
(0,	0.6,	0.6,	0)	0.0596	0.0348	0.0579	0.0480	0.0311	0.0508	0.0620	0.0371	0.0534	0.0513	0.0316	0.0507
(0,	0.6,	0.6,	$\frac{2}{\sqrt{n}}$)	0.3657	0.2867	0.3600	0.4042	0.3191	0.4019	0.3617	0.2775	0.3597	0.4052	0.3137	0.3980
(0,	0.6,	0.6,	$\frac{4}{\sqrt{n}}$)	0.9163	0.8760	0.9236	0.9423	0.9092	0.9389	0.9175	0.8782	0.9186	0.9387	0.9003	0.9332
(0,	1,	0.6,	0)	0.0583	0.0410	0.0538	0.0528	0.0359	0.0486	0.0593	0.0406	0.0545	0.0534	0.0323	0.0494
(0,	1,	0.6,	$\frac{2}{\sqrt{n}}$)	0.3498	0.3056	0.3582	0.4075	0.3460	0.4131	0.3575	0.3045	0.3567	0.4177	0.3483	0.4115
(0,	1,	0.6,	$\frac{4}{\sqrt{n}}$)	0.8400	0.8919	0.9132	0.8354	0.9195	0.9417	0.9214	0.8910	0.9151	0.9212	0.9261	0.9411
(0,	1,	1,	0)	0.0626	0.0391	0.0536	0.0497	0.0359	0.0505	0.0672	0.0421	0.0562	0.0529	0.0345	0.0560
(0,	1,	1,	$\frac{2}{\sqrt{n}}$)	0.3741	0.3097	0.3521	0.4169	0.3601	0.4120	0.3815	0.3050	0.3450	0.4207	0.3619	0.4170
(0,	1,	1,	$\frac{4}{\sqrt{n}}$)	0.8943	0.8928	0.9174	0.9267	0.9284	0.9476	0.8876	0.8964	0.9222	0.9238	0.9278	0.9436
(0.4,	0.6,	0.6,	0)	0.0571	0.0333	0.0577	0.0496	0.0304	0.0507	0.0573	0.0369	0.0545	0.0510	0.0330	0.0497
(0.4,	0.6,	0.6,	$\frac{2}{\sqrt{n}}$)	0.3660	0.3002	0.3632	0.4081	0.3361	0.4062	0.3732	0.3052	0.3627	0.4041	0.3423	0.4011
(0.4,	0.6,	0.6,	$\frac{4}{\sqrt{n}}$)	0.9112	0.8807	0.9155	0.9355	0.9082	0.9374	0.9070	0.8764	0.9141	0.9393	0.9086	0.9392
(0.4,	1,	0.6,	0)	0.0594	0.0421	0.0499	0.0511	0.0389	0.0531	0.0583	0.0437	0.0514	0.0513	0.0363	0.0496
(0.4,	1,	0.6,	$\frac{2}{\sqrt{n}}$)	0.3661	0.3273	0.3715	0.4209	0.3747	0.4243	0.3580	0.3309	0.3735	0.4215	0.3734	0.4178
(0.4,	1,	0.6,	$\frac{4}{\sqrt{n}}$)	0.8368	0.8986	0.9182	0.9233	0.9333	0.9485	0.8303	0.8974	0.9150	0.9250	0.9298	0.9443
(0.4,	1,	1,	0)	0.0678	0.0567	0.0520	0.0494	0.0535	0.0486	0.0657	0.0596	0.0513	0.0530	0.0511	0.0514
(0.4,	1,	1,	$\frac{2}{\sqrt{n}}$)	0.4387	0.4227	0.4246	0.4790	0.4782	0.4682	0.4390	0.4275	0.4127	0.4753	0.4727	0.4665
(0.4,	1,	1,	$\frac{4}{\sqrt{n}}$)	0.9156	0.9596	0.9607	0.9497	0.9720	0.9701	0.9181	0.9555	0.9574	0.9454	0.9738	0.9728
(0.8,	0.6,	0.6,	0)	0.0532	0.0089	0.0567	0.0538	0.0057	0.0495	0.0544	0.0074	0.0516	0.0502	0.0059	0.0531
(0.8,	0.6,	0.6,	$\frac{2}{\sqrt{n}}$)	0.2378	0.0703	0.2359	0.2577	0.0787	0.2535	0.2376	0.0677	0.2253	0.2560	0.075	0.2717
(0.8,	0.6,	0.6,	$\frac{4}{\sqrt{n}}$)	0.6951	0.4030	0.6907	0.7485	0.4426	0.7544	0.6938	0.3900	0.6909	0.7519	0.4463	0.7538
(0.8,	1,	0.6,	0)	0.0569	0.0088	0.0516	0.0525	0.0076	0.0487	0.0521	0.0102	0.0539	0.0498	0.0073	0.0512
(0.8,	1,	0.6,	$\frac{2}{\sqrt{n}}$)	0.2359	0.0747	0.2316	0.2676	0.0880	0.2744	0.2422	0.0787	0.2436	0.2745	0.0839	0.2684
(0.8,	1,	0.6,	$\frac{4}{\sqrt{n}}$)	0.6691	0.4528	0.7067	0.7700	0.4867	0.7752	0.6730	0.4425	0.7066	0.7623	0.4950	0.7653
(0.8,	1,	1,	0)	0.0616	0.0245	0.0550	0.0516	0.0201	0.0507	0.0604	0.0211	0.0587	0.0513	0.0193	0.0519
(0.8,	1,	1,	$\frac{2}{\sqrt{n}}$)	0.3501	0.2263	0.3254	0.3708	0.2424	0.3566	0.3445	0.2251	0.3293	0.3688	0.2348	0.3648
(0.8,	1,	1,	$\frac{4}{\sqrt{n}}$)	0.8719	0.8202	0.8896	0.8999	0.8254	0.9021	0.8679	0.8205	0.8917	0.8968	0.8266	0.9056

**Table 2:** Empirical sizes and powers of GARCH structural $U_{t}$.

(ρ,	ϕ,	A,	λ)	α=0						α=0.01					
				n=200			n=800			n=200			n=800		
				**PEL**	**PI**	**BG**	**PEL**	**PI**	**BG**	**PEL**	**PI**	**BG**	**PEL**	**PI**	**BG**
(0,	0.6,	0.6,	0)	0.0653	0.0647	0.1416	0.0560	0.0556	0.1632	0.0658	0.0622	0.1419	0.0542	0.0544	0.1622
(0,	0.6,	0.6,	$\frac{2}{\sqrt{n}}$)	0.3660	0.3813	0.5544	0.3794	0.3929	0.6183	0.3707	0.3796	0.5599	0.3734	0.4039	0.6146
(0,	0.6,	0.6,	$\frac{4}{\sqrt{n}}$)	0.7632	0.8689	0.9621	0.7591	0.8986	0.9759	0.7583	0.8702	0.9626	0.7594	0.9056	0.9754
(0,	1,	0.6,	0)	0.0658	0.0612	0.1508	0.0598	0.0526	0.1537	0.0656	0.0620	0.1386	0.0575	0.0520	0.1607
(0,	1,	0.6,	$\frac{2}{\sqrt{n}}$)	0.3486	0.3651	0.5393	0.3756	0.3983	0.6106	0.3391	0.3622	0.5406	0.3785	0.3811	0.6046
(0,	1,	0.6,	$\frac{4}{\sqrt{n}}$)	0.6795	0.8602	0.9516	0.7192	0.9018	0.9745	0.6867	0.8672	0.9595	0.7205	0.9079	0.9760
(0,	1,	1,	0)	0.0735	0.0625	0.1473	0.0593	0.0557	0.1631	0.074	0.0644	0.1462	0.0565	0.0547	0.1628
(0,	1,	1,	$\frac{2}{\sqrt{n}}$)	0.3847	0.3627	0.5322	0.3795	0.3876	0.6067	0.3943	0.3628	0.5384	0.3931	0.3917	0.6019
(0,	1,	1,	$\frac{4}{\sqrt{n}}$)	0.8440	0.8639	0.9536	0.7583	0.9049	0.9770	0.8441	0.8553	0.9570	0.7547	0.8957	0.9767
(0.4,	0.6,	0.6,	0)	0.0701	0.0436	0.1410	0.0531	0.0364	0.1524	0.0654	0.0428	0.1395	0.0525	0.038	0.1570
(0.4,	0.6,	0.6,	$\frac{2}{\sqrt{n}}$)	0.3449	0.2963	0.5106	0.3707	0.3024	0.5733	0.3458	0.2924	0.5083	0.3626	0.3064	0.5684
(0.4,	0.6,	0.6,	$\frac{4}{\sqrt{n}}$)	0.7459	0.7970	0.9408	0.7363	0.8424	0.9654	0.7414	0.8002	0.9412	0.7420	0.8442	0.9633
(0.4,	1,	0.6,	0)	0.0705	0.0437	0.1379	0.0629	0.0338	0.1526	0.0619	0.0456	0.1404	0.0634	0.0398	0.1631
(0.4,	1,	0.6,	$\frac{2}{\sqrt{n}}$)	0.3239	0.2936	0.4983	0.3598	0.3038	0.5659	0.3175	0.2895	0.4991	0.3626	0.3042	0.5616
(0.4,	1,	0.6,	$\frac{4}{\sqrt{n}}$)	0.6694	0.7881	0.9342	0.7100	0.8444	0.9620	0.6619	0.7910	0.9348	0.7104	0.8379	0.9608
(0.4,	1,	1,	0)	0.0792	0.0495	0.1408	0.0621	0.0426	0.1637	0.0769	0.0500	0.1389	0.0598	0.0401	0.1524
(0.4,	1,	1,	$\frac{2}{\sqrt{n}}$)	0.3608	0.2868	0.4990	0.3765	0.3153	0.5691	0.3714	0.2909	0.4915	0.3715	0.3173	0.5611
(0.4,	1,	1,	$\frac{4}{\sqrt{n}}$)	0.8265	0.7967	0.9373	0.7412	0.8532	0.9609	0.8196	0.7959	0.9344	0.7477	0.8532	0.9620
(0.8,	0.6,	0.6,	0)	0.0644	0.0028	0.1120	0.0513	0.0019	0.1370	0.0632	0.0020	0.1131	0.0584	0.0018	0.1364
(0.8,	0.6,	0.6,	$\frac{2}{\sqrt{n}}$)	0.2097	0.0266	0.3061	0.2104	0.0226	0.3634	0.2217	0.0269	0.2982	0.2116	0.0251	0.3609
(0.8,	0.6,	0.6,	$\frac{4}{\sqrt{n}}$)	0.5523	0.1972	0.6954	0.5658	0.1944	0.7821	0.5376	0.1961	0.6979	0.5608	0.1956	0.7789
(0.8,	1,	0.6,	0)	0.0654	0.0035	0.1147	0.0677	0.0029	0.1340	0.0670	0.0031	0.1174	0.0696	0.0021	0.1413
(0.8,	1,	0.6,	$\frac{2}{\sqrt{n}}$)	0.2050	0.0285	0.2935	0.2282	0.0235	0.3548	0.2089	0.0311	0.2983	0.2281	0.0227	0.3572
(0.8,	1,	0.6,	$\frac{4}{\sqrt{n}}$)	0.5008	0.1909	0.6842	0.5693	0.1922	0.7783	0.4951	0.1909	0.6875	0.5831	0.1941	0.7781
(0.8,	1,	1,	0)	0.0886	0.0030	0.1205	0.0701	0.0033	0.1344	0.0804	0.0034	0.1253	0.0717	0.0024	0.1379
(0.8,	1,	1,	$\frac{2}{\sqrt{n}}$)	0.2579	0.0333	0.2998	0.2469	0.0311	0.3683	0.2527	0.0316	0.2950	0.2440	0.0281	0.3679
(0.8,	1,	1,	$\frac{4}{\sqrt{n}}$)	0.6173	0.2238	0.7013	0.6097	0.2353	0.7940	0.6122	0.2156	0.7030	0.6139	0.2279	0.7993

**Table 3:** The results of PEL test in ARX fittings for stock returns.

Period	Obs.	Exo. Var.	Results for the Following Values of *q*							
			2	4	6	8	10	12	14	16
1927–2002	912	ldp	0.2727	0.1017	$0.0552{\;}^{*}$	$0.0447{\;}^{**}$	$0.0721{\;}^{*}$	$0.0981{\;}^{*}$	$0.0411{\;}^{**}$	$0.0733{\;}^{*}$
		lep	0.2692	$0.0962{\;}^{*}$	$0.0441{\;}^{**}$	$0.0345{\;}^{**}$	$0.0584{\;}^{*}$	$0.0857{\;}^{*}$	$0.0311{\;}^{**}$	$0.0573{\;}^{*}$
1927–1994	804	ldp	0.2145	$0.0538{\;}^{*}$	$0.0355{\;}^{**}$	$0.0326{\;}^{**}$	$0.0641{\;}^{*}$	$0.0886{\;}^{*}$	$0.0299{\;}^{**}$	$0.0460{\;}^{*}$
		lep	0.2317	$0.0528{\;}^{*}$	$0.0270{\;}^{**}$	$0.0248{\;}^{**}$	$0.0523{\;}^{*}$	$0.0823{\;}^{*}$	$0.0221{\;}^{**}$	$0.0335{\;}^{**}$
1952–2002	612	ldp	0.7848	0.8508	0.1528	0.2762	0.4311	0.4346	0.3533	0.4101
		lep	0.7850	0.8475	0.1466	0.2639	0.4222	0.4401	0.3397	0.3929
		tbl	0.7518	0.8740	0.1786	0.2986	0.4620	0.4849	0.4171	0.4522
		lty	0.7788	0.8742	0.1774	0.2948	0.4628	0.4989	0.4006	0.4401

Significance levels: * *p* < 0.1, ** *p* < 0.05.

**Table 4:** The results of diagnostic checking in ARX fittings for stock returns.

Period	Obs.	Exo. Var.	Test	Results for the Following Values of *q*							
				2	4	6	8	10	12	14	16
1927–2002	912	ldp, lep	PEL	0.5270	0.2376	$0.0976{\;}^{*}$	0.1018	0.1383	0.1858	$0.0801{\;}^{*}$	$0.0980{\;}^{*}$
			PI	0.9720	0.2891	0.1148	0.1034	0.1287	0.1722	$0.0892{\;}^{*}$	0.1086
			BG	$0.0320{\;}^{**}$	$0.0137{\;}^{**}$	$0.0017{\;}^{***}$	$0.0021{\;}^{***}$	$0.0011{\;}^{***}$	$0.0027{\;}^{***}$	$0.0004{\;}^{***}$	$0.0003{\;}^{***}$
1927–1994	804	ldp, lep	PEL	0.4398	0.1600	$0.0720{\;}^{*}$	$0.0805{\;}^{*}$	0.1359	0.1984	$0.0716{\;}^{*}$	$0.0755{\;}^{*}$
			PI	0.9958	0.2077	$0.0884{\;}^{*}$	$0.0848{\;}^{*}$	0.1310	0.1957	$0.0872{\;}^{*}$	$0.0885{\;}^{*}$
			BG	$0.0063{\;}^{***}$	$0.0054{\;}^{***}$	$0.0006{\;}^{***}$	$0.0008{\;}^{***}$	$0.0006{\;}^{***}$	$0.0016{\;}^{***}$	$0.0002{\;}^{***}$	$0.0001{\;}^{***}$
1952–2002	612	ldp, lep, tbl, lty	PEL	0.2836	0.4779	$0.0838{\;}^{*}$	0.1453	0.2582	0.2342	0.2476	0.2862
			PI	0.6568	0.8131	0.1910	0.3092	0.4592	0.4027	0.3940	0.4427
			BG	0.2187	0.4371	0.1084	0.2206	0.3437	0.3155	0.2825	0.3883

Significance levels: * *p* < 0.1, ** *p* < 0.05, *** *p* < 0.01.

**Table 5:** The results of diagnostic checking in ARX fittings for ozone concentration.

Period	Obs.	Test	Results for the Following Values of *q*							
			2	4	6	8	10	12	14	16
1 January–26 June	178	PEL	0.2817	$0.0562{\;}^{*}$	$0.0773{\;}^{*}$	$0.0738{\;}^{*}$	$0.0536{\;}^{*}$	$0.0499{\;}^{**}$	$0.0690{\;}^{*}$	$0.0231{\;}^{**}$
		PI	0.6555	0.1883	0.2412	0.3223	0.4602	0.3379	0.2664	0.1787
		BG	0.2939	0.1029	0.1773	0.2124	0.2545	0.3236	0.3637	0.3682
11 July–31 December	173	PEL	$0.0038{\;}^{***}$	$0.0021{\;}^{***}$	$0.0033{\;}^{***}$	$0.0074{\;}^{***}$	$0.0085{\;}^{***}$	$0.0075{\;}^{***}$	$0.0052{\;}^{***}$	$0.0011{\;}^{***}$
		PI	0.7063	0.9547	0.8998	0.8022	0.6021	0.5958	0.4126	0.2402
		BG	0.0352	$0.0686^{*}$	0.1856	0.2614	0.2499	0.3011	0.2450	0.2852

Significance levels: * *p* < 0.1, ** *p* < 0.05, *** *p* < 0.01.

## Data Availability

The data used in applications can be found in https://www.crsp.org and https://www.epa.gov (accessed on 27 July 2022).

## Appendix A. Proofs of the Main Results

In this appendix, we provide detailed proofs for the main results given in Section 2. Before proceeding further, we need to provide some necessary lemmas, which play key roles in proving Theorem 1. We denote $\theta_{0}$ and $\gamma_{0}$ as the true values of θ and γ, respectively. Note that we have $\gamma_{0}=0$ under the null hypothesis. With asymptotic equivalence, we do not distinguish the use of *n* and *N*.

**Lemma** **A1.**
*Under the same conditions of Theorem 1, when n→∞, we have*

(A1)
$$\begin{array}{cc} & \max_{t_{0}\leq t\leq n}\underset{B}{\sup}∥{\tilde{Z}}_{t}\left(\theta ,\gamma_{0}\right)∥=o_{p}\left(\sqrt{n}\right), \end{array}$$

(A2)
$$\begin{array}{cc} & \frac{1}{\sqrt{n}}\sum_{t=t_{0}}^{n}{\tilde{Z}}_{t}\left(\theta ,\gamma_{0}\right)=\frac{1}{\sqrt{n}}\sum_{t=t_{0}}^{n}{\tilde{Z}}_{t}\left(\theta_{0},\gamma_{0}\right)+O_{p}\left(1\right), \end{array}$$

(A3)
$$\begin{array}{cc} & \frac{1}{n}\sum_{t=t_{0}}^{n}{\tilde{Z}}_{t}\left(\theta ,\gamma_{0}\right){\tilde{Z}}_{t}^{⊤}\left(\theta ,\gamma_{0}\right)=\frac{1}{n}\sum_{t=t_{0}}^{n}{\tilde{Z}}_{t}\left(\theta_{0},\gamma_{0}\right){\tilde{Z}}_{t}^{⊤}\left(\theta_{0},\gamma_{0}\right)+o_{p}\left(1\right), \end{array}$$

*where $B=\{\theta :∥\theta -\theta_{0}∥<\frac{C}{\sqrt{n}}\}$ for some constant C>0.*

**Proof** **of Lemma A1.** We define $U_{t}=U_{t}\left(\theta_{0}\right)$ for short, where $t=t_{0},\cdots ,n$. To show the asymptotic result of (A1), we firstly expand the first element of ${\tilde{Z}}_{t}\left(\theta ,\gamma_{0}\right)$ as the following form:

$${\tilde{Z}}_{t,1}\left(\theta ,\gamma_{0}\right)=U_{t}-\left(\alpha -\alpha_{0}\right)-\left(\phi -\phi_{0}\right)Y_{t-1}-\sum_{i=1}^{p-1}\left(\psi_{i}-\psi_{i0}\right)\Delta Y_{t-i}-{\left(\beta -\beta_{0}\right)}^{⊤}X_{t-1},$$

so we can get that as n→∞,

$$\begin{array}{ccc} \max_{t_{0}\leq t\leq n}\underset{B}{\sup}\left|{\tilde{Z}}_{t,1}\left(\theta ,\gamma_{0}\right)\right| & \leq & \max_{t_{0}\leq t\leq n}|U_{t}|+O\left(\frac{1}{\sqrt{n}}\right)\times (\max_{t_{0}\leq t\leq n}\left|Y_{t-1}\right|\\ & & +\max_{t_{0}\leq t\leq n}\sum_{i=1}^{p-1}|\Delta Y_{t-i}|+\max_{t_{0}\leq t\leq n}∥X_{t-1}∥). \end{array}$$

By assumption **A4** and Markov inequality, $\max_{t_{0}\leq t\leq n}\left|U_{t}\right|=o_{p}\left(\sqrt{n}\right)$ is obtained. As for the items of $\max_{t_{0}\leq t\leq n}\left|Y_{t-1}\right|$, $\max_{t_{0}\leq t\leq n}\sum_{i=1}^{p-1}\left|\Delta Y_{t-i}\right|$ and $\max_{t_{0}\leq t\leq n}∥X_{t-1}∥$, we can also prove that they are of $o_{p}\left(\sqrt{n}\right)$. We take $\max_{t_{0}\leq t\leq n}\left|Y_{t-1}\right|$ as an example to display. For arbitrary ϵ>0 and some δ>0, as n→∞,

$$\begin{array}{ccc} P\left(\max_{t_{0}\leq t\leq n}\left|Y_{t-1}\right|\geq \epsilon \sqrt{n}\right) & \leq & \sum_{t=t_{0}}^{n}P\left(|Y_{t-1}|\geq \epsilon \sqrt{n}\right)\\ & \leq & \frac{1}{\epsilon^{2+\delta }n^{1+\delta /2}}E\left(\sum_{t=t_{0}}^{n}{\left|Y_{t-1}\right|}^{2+\delta }\right)\\ & \to & 0. \end{array}$$

The results of two other items are obtained similarly. Thus, $\max_{t_{0}\leq t\leq n}\sup_{B}\left|{\tilde{Z}}_{t,1}\left(\theta ,\gamma_{0}\right)\right|=o_{p}\left(\sqrt{n}\right)$. With this result, the further proofs of $\max_{t_{0}\leq t\leq n}\sup_{B}\left|{\tilde{Z}}_{t,j}\left(\theta ,\gamma_{0}\right)\right|=o_{p}\left(\sqrt{n}\right)$ for j=2,⋯,p+d+1 are trivial and similar; we omitted them. Meanwhile, those of the last *q* components are shown below. That is, when k=1,⋯,q,

$$\begin{array}{ccc} {\tilde{Z}}_{t,p+d+k+1}\left(\theta ,\gamma_{0}\right) & = & U_{t}\left(\theta \right)U_{t-k}\left(\theta \right)\\ & = & U_{t}U_{t-k}+U_{t}\left(U_{t-k}\left(\theta \right)-U_{t-k}\right)+\left(U_{t}\left(\theta \right)-U_{t}\right)U_{t-k}\\ & & +\left(U_{t}\left(\theta \right)-U_{t}\right)\left(U_{t-k}\left(\theta \right)-U_{t-k}\right)\\ & := & B_{1}+B_{2}+B_{3}+B_{4}, \end{array}$$

where $U_{t}\left(\theta \right)-U_{t}=\left(\alpha -\alpha_{0}\right)+\left(\phi -\phi_{0}\right)Y_{t-1}+\sum_{i=1}^{p-1}\left(\psi_{i}-\psi_{i0}\right)\Delta Y_{t-i}+{\left(\beta -\beta_{0}\right)}^{⊤}X_{t-1}$. The details of proving $\max_{t_{0}\leq t\leq n}\left|B_{i}\right|=o_{p}\left(\sqrt{n}\right)$ for i=1,2,3 are included in that for i=4, so we only expand $B_{4}$; that is,

$$\begin{array}{ccc} B_{4} & = & {\left(\alpha -\alpha_{0}\right)}^{2}+\left(\alpha -\alpha_{0}\right)\left(\phi -\phi_{0}\right)Y_{t-1}+\left(\alpha -\alpha_{0}\right)\sum_{i=1}^{p-1}\left(\psi_{i}-\psi_{i0}\right)\Delta Y_{t-i}\\ & & +\left(\alpha -\alpha_{0}\right){\left(\beta -\beta_{0}\right)}^{⊤}X_{t-1}+\left(\alpha -\alpha_{0}\right)\left(\phi -\phi_{0}\right)Y_{t-1}+{\left(\phi -\phi_{0}\right)}^{2}Y_{t-1}^{2}\\ & & +\left(\phi -\phi_{0}\right)Y_{t-1}\sum_{i=1}^{p-1}\left(\psi_{i}-\psi_{i0}\right)\Delta Y_{t-i}+\left(\phi -\phi_{0}\right)Y_{t-1}{\left(\beta -\beta_{0}\right)}^{⊤}X_{t-1}\\ & & +\left(\alpha -\alpha_{0}\right)\sum_{i=1}^{p-1}\left(\psi_{i}-\psi_{i0}\right)\Delta Y_{t-i}+\left(\phi -\phi_{0}\right)Y_{t-1}\sum_{i=1}^{p-1}\left(\psi_{i}-\psi_{i0}\right)\Delta Y_{t-i}\\ & & +{\left(\sum_{i=1}^{p-1}\left(\psi_{i}-\psi_{i0}\right)\Delta Y_{t-i}\right)}^{2}+{\left(\beta -\beta_{0}\right)}^{⊤}X_{t-1}\sum_{i=1}^{p-1}\left(\psi_{i}-\psi_{i0}\right)\Delta Y_{t-i}\\ & & +\left(\alpha -\alpha_{0}\right){\left(\beta -\beta_{0}\right)}^{⊤}X_{t-1}+\left(\phi -\phi_{0}\right)Y_{t-1}{\left(\beta -\beta_{0}\right)}^{⊤}X_{t-1}\\ & & +{\left(\beta -\beta_{0}\right)}^{⊤}X_{t-1}\sum_{i=1}^{p-1}\left(\psi_{i}-\psi_{i0}\right)\Delta Y_{t-i}+{\left(\beta -\beta_{0}\right)}^{⊤}X_{t-1}X_{t-1}^{⊤}\left(\beta -\beta_{0}\right). \end{array}$$

By the aforementioned techniques, we can find that the maximum of each term from *t* in $t_{0}$ to *n* in the expansion of $B_{4}$ is $o_{p}\left(\sqrt{n}\right)$. This finishes the proof of (A1). Next, for j=1,2,⋯,p+d+1 as n→∞, the proofs of

$$\begin{array}{c} \frac{1}{\sqrt{n}}\sum_{t=t_{0}}^{n}\left({\tilde{Z}}_{t,j}\left(\theta ,\gamma_{0}\right)-{\tilde{Z}}_{t,j}\left(\theta_{0},\gamma_{0}\right)\right)=O_{p}\left(1\right) \end{array}$$

are trivial. Thus, we only show the details of the last *q* components of (A2); that is, when k=1,⋯,q, we have

$$\begin{array}{ccc} & & \frac{1}{\sqrt{n}}\sum_{t=t_{0}}^{n}\left({\tilde{Z}}_{t,k}\left(\theta ,\gamma_{0}\right)-{\tilde{Z}}_{t,k}\left(\theta_{0},\gamma_{0}\right)\right)\\ & = & \frac{1}{\sqrt{n}}\sum_{t=t_{0}}^{n}\left(U_{t}\left(\theta \right)U_{t-k}\left(\theta \right)-U_{t}U_{t-k}\right)\\ & = & \frac{1}{\sqrt{n}}\sum_{t=t_{0}}^{n}\left(U_{t}\left(U_{t-k}\left(\theta \right)-U_{t-k}\right)+\left(U_{t}\left(\theta \right)-U_{t}\right)U_{t-k}+\left(U_{t}\left(\theta \right)-U_{t}\right)\left(U_{t-k}\left(\theta \right)-U_{t-k}\right)\right)\\ & = & \frac{1}{\sqrt{n}}\sum_{t=t_{0}}^{n}B_{2}+\frac{1}{\sqrt{n}}\sum_{t=t_{0}}^{n}B_{3}+\frac{1}{\sqrt{n}}\sum_{t=t_{0}}^{n}B_{4}\\ & := & C_{1}+C_{2}+C_{3}. \end{array}$$

Moreover, we expand $C_{1}$ as

$$\begin{array}{ccc} C_{1} & = & \frac{1}{\sqrt{n}}\left(\alpha -\alpha_{0}\right)\sum_{t=t_{0}}^{n}U_{t-k}+\frac{1}{\sqrt{n}}\left(\phi -\phi_{0}\right)\sum_{t=t_{0}}^{n}Y_{t-1}U_{t-k}\\ & & +\frac{1}{\sqrt{n}}\sum_{i=1}^{p-1}\left(\psi_{i}-\psi_{i0}\right)\sum_{t=t_{0}}^{n}\Delta Y_{t-i}U_{t-k}+\frac{1}{\sqrt{n}}\left(\beta -\beta_{0}\right)\sum_{t=t_{0}}^{n}X_{t-1}U_{t-k}\\ & = & O_{p}\left(1\right). \end{array}$$

Similarly, we can show that $C_{2},C_{3}=O_{p}\left(1\right)$ as n→∞. By then, we have finished the proof of (A2). The proof of (A3) contains no new questionable part that need to be discussed, so we omit it as an analogue of (A2). □

**Lemma** **A2.**
*Under the same conditions of Theorem 1 with $H_{0}$ holding, when n→∞, we have*

(A4)
$$\begin{array}{c} \frac{1}{\sqrt{n}}\sum_{t=t_{0}}^{n}{\tilde{Z}}_{t}\left(\theta_{0},0\right)\overset{d}{⟶}N\left(0,\Sigma \right), \end{array}$$

(A5)
$$\begin{array}{c} \frac{1}{n}\sum_{t=t_{0}}^{n}{\tilde{Z}}_{t}\left(\theta_{0},0\right){\tilde{Z}}_{t}^{⊤}\left(\theta_{0},0\right)\overset{p}{⟶}\Sigma , \end{array}$$

*where $\overset{d}{⟶}$ denotes convergence in distribution, and $\overset{p}{⟶}$ denotes convergence in probability. Matrix Σ is defined as $E\left({\tilde{Z}}_{t}\left(\theta_{0},0\right){\tilde{Z}}_{t}^{⊤}\left(\theta_{0},0\right)\right)$. Note that ***Σ*** is a positive definite matrix.*

**Proof** **of Lemma A2.** According to assumption **A2**, it is easy to check that ${\left\{{\tilde{Z}}_{t}\left(\theta ,\gamma \right)\right\}}_{t=t_{0}}^{n}$ is a Martingale difference series with respect to $F_{t-1}$. Hence, based on assumptions **A1**–**A5**, we can further show (A4) by the Martingale central limit theorem in [35] and the Cramér–Wold device. The proof of (A5) is trivial based on (A4); we omit the details. □

**Proof** **of Theorem 1.** Based on the results obtained in Lemma A1 and Lemma A2, we can finally prove the asymptotic chi-squared distribution of our proposed test statistics. As shown in [36], the solution of $p_{t}$ by Lagrange multipliers is

$$p_{t}=\frac{1}{n}\frac{1}{1+\lambda^{⊤}{\tilde{Z}}_{t}\left(\theta ,\gamma_{0}\right)},\;\;t=t_{0},\cdots ,n.$$

Note that the estimating equations require that $\sum_{t=t_{0}}^{n}p_{t}{\tilde{Z}}_{t}\left(\theta ,\gamma_{0}\right)=0$. Denote that

$$g\left(\theta ,\gamma ,x\right):=\frac{1}{n}\sum_{t=t_{0}}^{n}\frac{{\tilde{Z}}_{t}\left(\theta ,\gamma \right)}{1+x^{⊤}{\tilde{Z}}_{t}\left(\theta ,\gamma \right)},\;\mathrm{and}$$

$$\xi_{t}\left(\theta ,\gamma_{0}\right):=\lambda^{⊤}{\tilde{Z}}_{t}\left(\theta ,\gamma_{0}\right),$$

where λ is the solution of g(θ,γ,x)=0 with respect to x for fixed θ. Firstly, we show that $\left||\lambda |\right|=O_{p}\left(\frac{1}{\sqrt{n}}\right)$ as n→∞. Define v from λ=ρv with ρ=||λ||. We obtain the following results from $∥g(\theta ,\gamma_{0},\lambda )∥=0$. Assume θ∈B; we then have

$$\begin{array}{ccc} 0 & = & \left|\lambda^{⊤}g\left(\theta ,\gamma_{0},\lambda \right)\right|\\ & = & \left|\frac{1}{n}\sum_{t=t_{0}}^{n}\xi_{t}\left(\theta ,\gamma_{0}\right)-\frac{1}{n}\sum_{t=t_{0}}^{n}\frac{\xi_{t}^{2}\left(\theta ,\gamma_{0}\right)}{1+\xi_{t}\left(\theta ,\gamma_{0}\right)}\right|\\ & \geq & \left|\frac{1}{n}\sum_{t=t_{0}}^{n}\frac{\xi_{t}^{2}\left(\theta ,\gamma_{0}\right)}{1+\xi_{t}\left(\theta ,\gamma_{0}\right)}\right|-\left|\frac{1}{n}\sum_{t=t_{0}}^{n}\xi_{t}\left(\theta ,\gamma_{0}\right)\right|\\ & \geq & \frac{1}{n}\sum_{t=t_{0}}^{n}\frac{\xi_{t}^{2}\left(\theta ,\gamma_{0}\right)}{1+\max_{t_{0}\leq t\leq n}\xi_{t}\left(\theta ,\gamma_{0}\right)}-\left|\frac{1}{n}\sum_{t=t_{0}}^{n}\xi_{t}\left(\theta ,\gamma_{0}\right)\right|. \end{array}$$

Denote that $D_{n}\left(\theta \right)=\frac{1}{\sqrt{n}}\sum_{t=t_{0}}^{n}{\tilde{Z}}_{t}\left(\theta ,\gamma_{0}\right)$, $F_{n}\left(\theta \right)=\frac{1}{n}\sum_{t=t_{0}}^{n}{\tilde{Z}}_{t}\left(\theta ,\gamma_{0}\right){\tilde{Z}}_{t}^{⊤}\left(\theta ,\gamma_{0}\right)$; then, the above inequality could be written as

$$\begin{array}{ccc} \rho^{2}v^{⊤}F_{n}\left(\theta \right)v & \leq & \left|\frac{1}{n}\sum_{t=t_{0}}^{n}\xi_{t}\left(\theta ,\gamma_{0}\right)\right|\left(1+\max_{t_{0}\leq t\leq n}\xi_{t}\left(\theta ,\gamma_{0}\right)\right)\\ & \leq & \left(\left|\frac{\rho }{n}\sum_{t=t_{0}}^{n}v^{⊤}{\tilde{Z}}_{t}\left(\theta_{0},\gamma_{0}\right)\right|+\left|\frac{\rho }{n}\sum_{t=t_{0}}^{n}v^{⊤}\left({\tilde{Z}}_{t}\left(\theta ,\gamma_{0}\right)-{\tilde{Z}}_{t}\left(\theta_{0},\gamma_{0}\right)\right)\right|\right)\\ & & \times \left(1+\left|\rho \right|\max_{t_{0}\leq t\leq n}\left|v^{⊤}{\tilde{Z}}_{t}\left(\theta ,\gamma_{0}\right)\right|\right)\\ & = & \left|\rho \right|\times O_{p}\left(\frac{1}{\sqrt{n}}\right)+\rho^{2}\times O_{p}\left(\frac{1}{\sqrt{n}}\right)\times o_{p}\left(\sqrt{n}\right). \end{array}$$

Note that $F_{n}\left(\theta \right)\overset{p}{⟶}\Sigma$, and Σ is a positive definite matrix. Thus, $v^{⊤}F_{n}\left(\theta \right)v$ is greater than the minimum eigenvalue of $F_{n}\left(\theta \right)=\Sigma +o_{p}\left(1\right)$. This finishes $\left||\lambda |\right|=O_{p}\left(\frac{1}{\sqrt{n}}\right)$. Next, we show that for θ∈B,

(A6) $$\lambda =\frac{1}{\sqrt{n}}\left(\Sigma^{-1}D_{n}\left(\theta \right)+o_{p}\left(1\right)\right).$$

According to $\sum_{t=t_{0}}^{n}p_{t}{\tilde{Z}}_{t}\left(\theta ,\gamma_{0}\right)=0$, we have

$$\begin{array}{ccc} 0 & = & g(\theta ,\gamma_{0},\lambda )\\ & = & \frac{1}{n}\sum_{t=t_{0}}^{n}\left({\tilde{Z}}_{t}\left(\theta ,\gamma \right)\frac{1-\xi_{t}^{2}\left(\theta ,\gamma \right)+\xi_{t}^{2}\left(\theta ,\gamma \right)}{1+\xi_{t}\left(\theta ,\gamma \right)}\right)\\ & = & \frac{1}{n}\sum_{t=t_{0}}^{n}\left({\tilde{Z}}_{t}\left(\theta ,\gamma \right)\left(1-\xi_{t}\left(\theta ,\gamma \right)\right)\right)+\frac{1}{n}\sum_{t=t_{0}}^{n}\left({\tilde{Z}}_{t}\left(\theta ,\gamma \right)\frac{\xi_{t}^{2}\left(\theta ,\gamma \right)}{1+\xi_{t}\left(\theta ,\gamma \right)}\right)\\ & = & \frac{1}{\sqrt{n}}D_{n}\left(\theta \right)+\Sigma \lambda +\frac{1}{n}\sum_{t=t_{0}}^{n}\left({\tilde{Z}}_{t}\left(\theta ,\gamma \right)\frac{\xi_{t}^{2}\left(\theta ,\gamma \right)}{1+\xi_{t}\left(\theta ,\gamma \right)}\right). \end{array}$$

As discussed above, we have

$$\max_{t_{0}\leq t\leq n}\xi_{t}\left(\theta ,\gamma_{0}\right)\leq \left|\rho \right|\max_{t_{0}\leq t\leq n}\left|v^{⊤}{\tilde{Z}}_{t}\left(\theta ,\gamma_{0}\right)\right|=O_{p}\left(\frac{1}{\sqrt{n}}\right)\times o_{p}\left(\sqrt{n}\right)=o_{p}\left(1\right).$$

Then we can further get that

$$\begin{array}{ccc} & & ∥\frac{1}{n}\sum_{t=t_{0}}^{n}\left({\tilde{Z}}_{t}\left(\theta ,\gamma_{0}\right)\frac{\xi_{t}^{2}\left(\theta ,\gamma_{0}\right)}{1+\xi_{t}\left(\theta ,\gamma_{0}\right)}\right)∥\\ & = & ∥\lambda^{⊤}\frac{1}{n}\sum_{t=t_{0}}^{n}\left({\tilde{Z}}_{t}\left(\theta ,\gamma_{0}\right){\tilde{Z}}_{t}^{⊤}\left(\theta ,\gamma_{0}\right)\frac{\xi_{t}\left(\theta ,\gamma_{0}\right)}{1+\xi_{t}\left(\theta ,\gamma_{0}\right)}\right)∥\\ & = & O_{p}\left(\frac{1}{\sqrt{n}}\right)\times o_{p}\left(1\right)=o_{p}\left(\frac{1}{\sqrt{n}}\right) \end{array}$$

This result uses the positive definiteness of $F_{n}\left(\theta \right)$ again. Here, we have proved (A6). Finally, we prove the limiting distribution of our proposed statistics. We expand $-2\log\tilde{L}\left(\theta ,\gamma_{0}\right)$ by Taylor expansion, where $\zeta_{t}$ denotes some point between 0 and $\xi_{t}\left(\theta ,\gamma_{0}\right)$.

$$\begin{array}{ccc} & & -2\log\tilde{L}\left(\theta ,\gamma_{0}\right)\\ & = & 2\sum_{t=t_{0}}^{n}\xi_{t}\left(\theta ,\gamma_{0}\right)-\sum_{t=t_{0}}^{n}\xi_{t}^{2}\left(\theta ,\gamma_{0}\right)+\sum_{t=t_{0}}^{n}\frac{2}{3{\left(1+\zeta_{t}\right)}^{3}}\xi_{t}^{3}\left(\theta ,\gamma_{0}\right)\\ & = & 2\sqrt{n}\lambda^{⊤}\left(\frac{1}{\sqrt{n}}\sum_{t=t_{0}}^{n}{\tilde{Z}}_{t}\left(\theta ,\gamma_{0}\right)\right)-n\lambda^{⊤}\left(\frac{1}{n}\sum_{t=t_{0}}^{n}{\tilde{Z}}_{t}\left(\theta ,\gamma_{0}\right){\tilde{Z}}_{t}^{⊤}\left(\theta ,\gamma_{0}\right)\right)\lambda \\ & & +\sum_{t=t_{0}}^{n}\frac{2}{3{\left(1+\zeta_{t}\right)}^{3}}\xi_{t}^{3}\left(\theta ,\gamma_{0}\right)\\ & = & 2\left(D_{n}^{⊤}\left(\theta \right)\Sigma^{-1}+o_{p}\left(1\right)\right)D_{n}\left(\theta \right)-\left(D_{n}^{⊤}\left(\theta \right)\Sigma^{-1}+o_{p}\left(1\right)\right)F_{n}\left(\theta \right)\left(D_{n}\left(\theta \right)\Sigma^{-1}+o_{p}\left(1\right)\right)\\ & & +\sum_{t=t_{0}}^{n}\frac{2}{3{\left(1+\zeta_{t}\right)}^{3}}\xi_{t}^{3}\left(\theta ,\gamma_{0}\right)\\ & = & D_{n}^{⊤}\left(\theta \right)\Sigma^{-1}D_{n}\left(\theta \right)+o_{p}\left(1\right) \end{array}$$

Notice that for some constant C>0,

$$\begin{array}{ccc} \left|\sum_{t=t_{0}}^{n}\frac{2}{3{\left(1+\zeta_{t}\right)}^{3}}\xi_{t}^{3}\left(\theta ,\gamma_{0}\right)\right| & \leq & C\max_{t_{0}\leq t\leq n}\xi_{t}\left(\theta ,\gamma_{0}\right)\sum_{t=t_{0}}^{n}\xi_{t}^{2}\left(\theta ,\gamma_{0}\right)\\ & = & C\max_{t_{0}\leq t\leq n}\xi_{t}\left(\theta ,\gamma_{0}\right)\sum_{t=t_{0}}^{n}\lambda^{⊤}F_{n}\left(\theta \right)\lambda \\ & = & o_{p}\left(1\right). \end{array}$$

Assume θ∈B and define matrix $B_{n}\left(\theta \right)$ as the solution of

$$D_{n}\left(\theta \right)-D_{n}\left(\theta_{0}\right)=B_{n}\left(\theta \right)\left(\theta -\theta_{0}\right).$$

Notice that $B_{n}\left(\theta \right)=\left(\begin{array}{c} B_{n}^{☆}\left(\theta \right)\\ 0_{q\times (p+d+q+1)} \end{array}\right)$, $0_{x\times y}$ denotes a zero matrix of order x×y. Assume that $\tilde{\theta }$ is the solution of the following objective function; that is, $\tilde{\theta }:=\arg\min_{B}G\left(\theta \right)$ with

$$\begin{array}{ccc} G(\theta ) & = & -2\log\tilde{L}\left(\theta ,\gamma_{0}\right)+2\log\tilde{L}\left(\theta_{0},\gamma_{0}\right)\\ & = & D_{n}^{⊤}\left(\theta \right)\Sigma^{-1}D_{n}\left(\theta \right)-D_{n}^{⊤}\left(\theta_{0}\right)\Sigma^{-1}D_{n}\left(\theta_{0}\right)+o_{p}\left(1\right)\\ & = & {\left(\theta -\theta_{0}\right)}^{⊤}B_{n}^{⊤}\left(\theta \right)\Sigma^{-1}B_{n}\left(\theta \right)\left(\theta -\theta_{0}\right)+2{\left(\theta -\theta_{0}\right)}^{⊤}B_{n}^{⊤}\left(\theta \right)\Sigma^{-1}D_{n}\left(\theta_{0}\right)+o_{p}\left(1\right). \end{array}$$

From [36], we know that $\inf_{B}G\left(\theta \right)=G\left(\theta_{0}\right)=0$. Solving G(θ)=0, we can obtain

$$\left(\tilde{\theta }-\theta_{0}\right)=-{\left(B_{n}^{⊤}\left(\tilde{\theta }\right)\Sigma^{-1}B_{n}\left(\tilde{\theta }\right)\right)}^{-1}\Sigma^{-1}D_{n}\left(\theta_{0}\right)+o_{p}\left(1\right).$$

Consequently, we can show the asymptotic result of the proposed log EL statistic. Assuming n→∞, we have

$$\begin{array}{ccc} & & -2\log\tilde{L}\left(\tilde{\theta },\gamma_{0}\right)\\ & = & D_{n}^{⊤}\left(\tilde{\theta }\right)\Sigma^{-1}D_{n}\left(\tilde{\theta }\right)+o_{p}\left(1\right)\\ & = & {\left(B_{n}\left(\tilde{\theta }\right)\left(\tilde{\theta }-\theta_{0}\right)+D_{n}\left(\theta_{0}\right)\right)}^{⊤}\Sigma^{-1}\left(B_{n}\left(\tilde{\theta }\right)\left(\tilde{\theta }-\theta_{0}\right)+D_{n}\left(\theta_{0}\right)\right)+o_{p}\left(1\right)\\ & = & {\left(\Sigma^{-\frac{1}{2}}D_{n}\left(\tilde{\theta }\right)\right)}^{⊤}\left(I_{p+d+q+1}-\Sigma^{\frac{1}{2}}B_{n}\left(\tilde{\theta }\right){\left(B_{n}^{⊤}\left(\tilde{\theta }\right)\Sigma^{-1}B_{n}\left(\tilde{\theta }\right)\right)}^{-1}B_{n}^{-1}\left(\tilde{\theta }\right)\Sigma^{\frac{1}{2}}\right)\\ & & \left(\Sigma^{-\frac{1}{2}}D_{n}\left(\tilde{\theta }\right)\right)+o_{p}\left(1\right)\\ & = & {\left(\Sigma^{-\frac{1}{2}}D_{n}\left(\tilde{\theta }\right)\right)}^{⊤}\left(\begin{array}{cc} 0_{\left(p+d+1)\times (p+d+1\right)} & 0_{(p+d+1)\times q}\\ 0_{(p+d+1)\times q} & I_{q} \end{array}\right)\left(\Sigma^{-\frac{1}{2}}D_{n}\left(\tilde{\theta }\right)\right)+o_{p}\left(1\right)\overset{d}{⟶}\chi_{q}^{2}. \end{array}$$

□
